# Splice modulating oligomers as cancer therapeutics

**DOI:** 10.18632/genesandcancer.222

**Published:** 2022-08-26

**Authors:** KuanHui E. Chen, Ameae M. Walker

**Keywords:** prolactin receptors, T regulatory cells, tumor microenvironment, receptor isoforms, dominant negatives

Genes are transcribed to produce pre-mRNAs, which are then spliced to create the mature mRNAs translated into protein. In recent years, improved deep sequencing technologies have shown greater than 90% of human pre-mRNAs undergo alternative splicing, thereby amplifying the potential protein products from each gene [1]. Alternatively spliced forms of pre-mRNA may code for proteins with related, distinct, or even opposing functions [1].

Many growth factor and hormone receptors and signaling molecules implicated in cancer have natural splice variants, some of which have been shown to act as dominant negatives. We hypothesized that by altering splicing to decrease growth-promoting and/or increase expression of dominant negative varieties we could eliminate abnormal dependence on growth factors, decrease metastatic potential, and promote cancer cell death.

By binding to specific intronic or exonic regions or intron-exon junctions, splice modulating oligomers, which are cDNA sequences, can alter the outcome of splicing [e.g., 2, 3]. To our knowledge, no one had previously tapped the potential of splice modulating oligomers to increase the relative activity of natural dominant negatives in order to combat disease. Where splice modulating oligomers had begun to be explored as therapeutics was for diseases that result from splicing errors and the production of a non-functional protein [4, 5].

Dominant negative receptors may inhibit signaling from the growth-promoting form of the receptor in a variety of ways. In the simplest situation, a dominant negative receptor binds ligand and therefore reduces availability to the growth-promoting receptor. In other instances, the dominant negative receptor may generate an alternate intracellular signal [e.g., 6–9]. Such amplification of the effect of dominant negative receptors through a signaling cascade makes an increase in their relative expression all the more effective. Importantly and additionally, the signals generated can promote differentiation and/or apoptotic cell death [6–8], thereby raising the possibility of greater effect, or even elimination of tumor cells.

As our proof of principle, we have applied splice modulating DNA oligomers to knockdown the proliferation-promoting and antiapoptotic long isoform of the prolactin receptor (LFPRLR), while still allowing signaling from the short, differentiation-promoting and pro-apoptotic forms. Although prolactin signaling is important to initiation and progression of prostate, ovarian, breast and blood cancers, our initial focus has been on breast cancer. Thus, we have tested the efficacy of splice modulating oligomers against highly aggressive, metastatic breast cancers in both syngeneic and human xenograft situations [10, 11]. Knockdown of the LFPRLR markedly decreased metastatic spread in both models [10]. However, only in the presence of an intact immune system did knockdown have a significant effect on survival [11]. Although not a totally surprising finding, it is yet one more example showing that assessment of future clinical treatment potential should be conducted in the presence of adaptive immunity.

**Figure 1:**
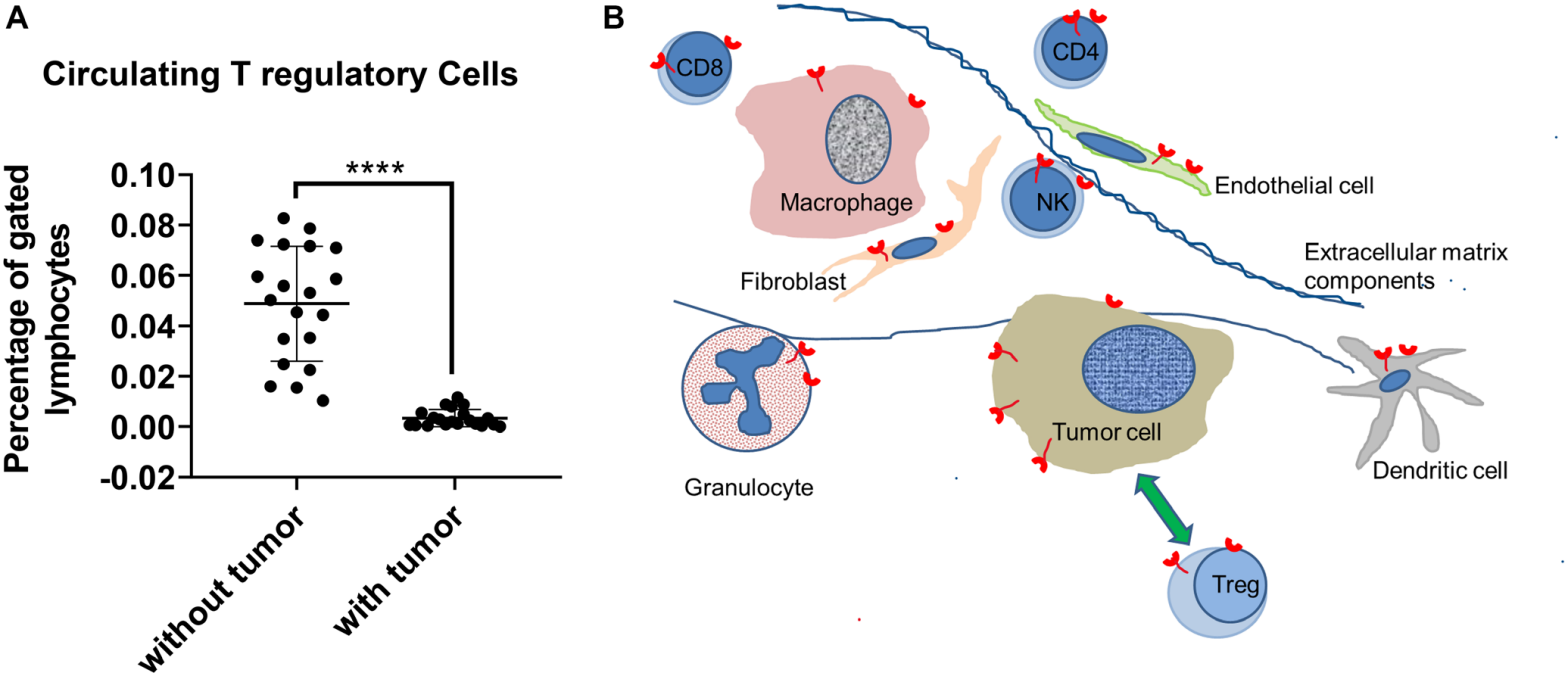
Tumor-T regulatory cell interactions. Syngeneic, triple negative mouse breast tumors attract T regulatory cells (Treg) out of the circulation (**A**) into the complex microenvironment of the tumor (**B**). All cell types in the tumor can express PRLR (shown in red), but some only when/if activated. The cells constituting the tumor parenchyma overexpress the long form of the PRLR (LF PRLR, shown in red with a longer intracellular portion) which promotes proliferation and survival. These parenchymal cells (and possibly other cells) produce CCL17, which attracts the Tregs from the circulation. Tregs suppress the function of anti-tumor effector cells (CD8+, CD4+, NK) and promote expression of mesenchymal genes in the parenchymal cells, thereby facilitating metastatic spread. Knockdown of the LF PRLR has differentiation and apoptotic promoting effects on the tumor parenchyma, amplified through the relative increase in the short forms of the PRLR, and reduces recruitment of Tregs, allowing activation of effector cells [10, 11]. Each dot in panel A represents an individual animal; ^****^p = 5.8 × 10^−8^; the green arrow represents the reciprocal influences between tumor cells and Tregs.

Accumulation of tumor T regulatory cells (Tregs) is associated with a worse prognosis in most cancer types. Orthotopic, syngeneic tumors of mouse triple negative breast cancer cells are so attractive to Tregs that one can demonstrate Treg depletion from the circulation (Figure 1A). Systemic knockdown of the LFPRLR increased the capacity for anti-tumor immunity through increased numbers of effector T cells [10, 11] and reduced numbers of Tregs in the tumor, the latter achieved through reduced tumor production of the recruiting chemokine, CCL17 [11]. The splice modulating oligomer also reduced both the metastasis-promoting ability of the Tregs still present in the tumor, and likely also their local intra-tumor suppressive function [11]. Some effects of LFPRLR knockdown on tumor parenchyma were direct while others were indirect via Tregs and *vice versa* (Figure 1B). Moreover, some effects of signaling through the LFPRLR on tumor parenchyma were opposite in the absence vs. presence of Tregs [11].

Therapeutics targeting Tregs are proving to be effective treatments for many cancers. However, because they target all Tregs, current immunotherapeutics can result in development of a variety of inflammatory disorders and autoimmune diseases [discussed in 11]. Even though all Tregs constitutively express LFPRLRs, the LFPRLRs in Tregs are normally resistant to knockdown, most likely because a splicing regulator occupies the target site of the splice modulating oligomer. Therefore, there was no negative effect on peripheral Treg function [11]. However, not only were there far fewer Tregs in the tumor with systemic LFPRLR knockdown, but the intra-tumoral environment and cellular crosstalk was altered such that the tumor Tregs showed knockdown of the LFPRLR and components of the immune synapse [11]. This finding illustrates the importance of examining therapeutic responses in the complex microenvironment of a tumor* in vivo *where local cytokines/chemokines can influence the outcome.

Adding to their attractiveness as therapeutics, splice modulating oligomers can be combined (or sequenced) to target several different transcripts, potentially increasing the impact of treatment, and likely making it more difficult for tumor cells to become resistant. In addition, as morpholino DNA oligomers they are more stable than short interfering or antisense RNAs and therefore do not require carrier technologies. They can be derivatized in a number of ways to promote cell penetration; in our studies, we used octaguanidine dendrimer derivatization and have shown very good knockdown in multiple tissues with no discernible toxicity at effective anti-tumor doses [10, 11]. In addition, as morpholino oligomers, they do not elicit an immune response to extracellular or abnormally localized intracellular DNA. Finally, by changing the ratio of normal splice variants, there should be no activation of the unfolded/misfolded protein stress response in cells. While these responses within cancer cells themselves can be beneficial for cancer treatment, unfolded protein responses in unintended targets can produce deleterious side effects.
